# Na^+^ compartmentalization related to salinity stress tolerance in upland cotton (*Gossypium hirsutum*) seedlings

**DOI:** 10.1038/srep34548

**Published:** 2016-10-04

**Authors:** Zhen Peng, Shoupu He, Junling Sun, Zhaoe Pan, Wenfang Gong, Yanli Lu, Xiongming Du

**Affiliations:** 1 State Key Laboratory of Cotton Biology/Institute of Cotton Research, Chinese Academy of Agricultural Sciences, Anyang, Henan 455000 China; 2 Maize Research Institute of Sichuan Agricultural University/Key Laboratory of Biology and Genetic Improvement of Maize in the Southwest Region, Ministry of Agriculture, Wenjiang, Sichuan 611130 China

## Abstract

The capacity for ion compartmentalization among different tissues and cells is the key mechanism regulating salt tolerance in plants. In this study, we investigated the ion compartmentalization capacity of two upland cotton genotypes with different salt tolerances under salt shock at the tissue, cell and molecular levels. We found that the leaf glandular trichome could secrete more salt ions in the salt-tolerant genotype than in the sensitive genotype, demonstrating the excretion of ions from tissue may be a new mechanism to respond to short-term salt shock. Furthermore, an investigation of the ion distribution demonstrated that the ion content was significantly lower in critical tissues and cells of the salt-tolerant genotype, indicating the salt-tolerant genotype had a greater capacity for ion compartmentalization in the shoot. By comparing the membrane H^+^-ATPase activity and the expression of ion transportation-related genes, we found that the H^+^-ATPase activity and Na^+^/H^+^ antiporter are the key factors determining the capacity for ion compartmentalization in leaves, which might further determine the salt tolerance of cotton. The novel function of the glandular trichome and the comparison of Na^+^ compartmentalization between two cotton genotypes with contrasting salt tolerances provide a new understanding of the salt tolerance mechanism in cotton.

The accumulation of excess salts in soil is a serious environmental problem that could adversely affect plant growth, geographical distribution, and crop productivity^1^,^2^. Soil salinity is more severe in arid and semi-arid regions and also occurs extensively in sub-humid and irrigated agricultural areas^3^. Wang *et al*. reported that the total area of saline soil in China is approximately 3.6 × 10^7^ ha, representing 4.88% of the country’s total available irrigated lands^4^. High salt levels mainly cause osmotic stress, ion toxicity and mineral perturbations (such as those of K^+^ and Ca^2+^) in plants^5^,^6^. Therefore, major research areas include the elucidation of salt tolerance mechanisms and the development of salt-resistant crops, which have attracted the interest of breeders and scientists in recent years^7^,^8^,^9^.

The key mechanisms of salt tolerance are the ability of plants to regulate Na^+^ uptake from soil to root, long-distance Na^+^ transport and intracellular Na^+^ compartmentalization in leaf cells and to secrete Na^+^ from leaves. Generally, compared with glycophytes, halophytes have greater control over Na^+^ influx, a stronger ability to coordinate the distribution of Na^+^ to various tissues, and a more efficient sequestration of Na^+^ into vacuoles^10^,^11^. Halophytes have evolved unique mechanisms, such as salt glands, bladders, and succulence, to excrete Na^+^ from their organizational structures^12^,^13^,^14^,^15^. In addition, these plants impart salt tolerance mechanisms that are coordinated between tissues and cells and mediated by membrane-related cation channels and transporters (tonoplasts and the plasmalemma Na^+^/H^+^ antiporter)^16^,^17^,^18^,^19^. Salt tolerance in plants refers to the ability to regulate intracellular Na^+^ homeostasis to minimize the cytotoxic effects of the ion and achieve osmotic adjustment^6^.

Salt tolerance mechanisms are different in different plants. Na^+^ and Cl^−^ are generally retained in the roots of barley, wheat, maize and sorghum under salt stress. However, this situation is different in upland cotton (*Gossypium hirsutum* L.)^11^. Cotton is a moderately salt-tolerant crop, but its salt tolerance threshold is no more than 7.7 ds·m^−1 ^20. Under salt stress, the growth of cotton is severely suppressed, especially at germination and the young seedling stage^20^,^21^. At the tissue level, cotton accumulates more than 95% of Na^+^ in the shoots^22^, although an earlier study reported no clear correlation between salt tolerance and Na^+^ accumulation in cotton^23^. Leidi & Saiz^24^ and Sun & Liu^25^ reported that the roots and leaves of some salt-tolerant cotton varieties could retain Na^+^.

In many halophytes, another important salt resistance mechanism is salt secretion, which regulates salt tolerance by secreting salt (especially NaCl) through salt glands in the leaves and by modulating the internal ion concentrations to a lower level. Some other salt secretory structures also exist, such as cellular glands in grass^26^, salt-secreting microhairs in halophytic wild rice^27^, glandular trichomes (GTs) in the leaves and stems of wild soybean^28^,^29^, glandular hairs in maize^30^, and hydathodes^31^. GTs (referred to as glands) on the surface of cotton leaves secrete various substances, e.g., cations, lipids, hydrous and hydrated carbonates, etc.^32^. However, few studies have investigated GTs further. Thus, it is unknown whether the GTs on the surface of cotton leaves can secrete NaCl.

An unreported phenomenon was discovered during our previous cotton germplasm salt tolerance test. We observed white salt crystals that emerged on the surface of cotton seedling leaves (both cotyledons and true leaves) after 24 h of exposure to NaCl solution with different salinity concentrations (2–6%, w/v) in soil under glasshouse conditions (the temperature was 20–32 °C) (Fig. 1A–D and Supplementary Fig. S1). Furthermore, we found differences in the amount of ion secretion among the different salt-tolerant lines. This phenomenon implied that cotton plants might have a mechanism for secreting ions from the leaves. In this study, we investigated the Na^+^ compartmentalization of two genotypes contrasting in salt tolerance under short-term salt shock at different tissue, cellular and molecular levels. Additionally, the role of GTs during Na^+^ compartmentalization and their contribution to salt tolerance were discussed.

## Results

### Na^+^ and K^+^ content *in vivo* and *in vitro* at the tissue level in salt-sensitive and -tolerant genotypes

Most of the true leaves of the salt-sensitive genotype Nandanbadidahua (NH) seedlings had visibly dried after 72 h of salt treatment, but they dried to a lesser degree than did those of the salt-tolerant genotype Earlistaple 7 (E7) seedlings (Fig. 2B). The relative changes in the Na^+^ and K^+^ contents in the roots, hypocotyls, cotyledons, stems, old leaves, and new leaves of the E7 and NH seedlings at six time points of NaCl shock are shown in Fig. 2C and Supplementary Fig. S2. The Na^+^ content rapidly increased in a time-dependent manner once the seedlings were transferred into a 200-mM NaCl solution, although the content was very low before NaCl treatment (0 h) in all samples. The roots of the salt-tolerant genotype (E7) accrued more Na^+^ than did the roots of the sensitive genotype (NH), whereas E7 contained a significantly lower level of Na^+^ in the new leaves at 24 and 72 h. In the old leaves and stems, Na^+^ retention was observed in NH, which exhibited a higher Na^+^ content than E7. However, there were no differences in the hypocotyls and cotyledons between the tolerant and sensitive genotypes. These results suggest that the higher Na^+^ absorption capacity of the roots and the lower level of Na^+^ in leaves of salt-tolerant genotypeE7 might prevent excessive Na^+^ from affecting shoot growth.

**Figure 1:**
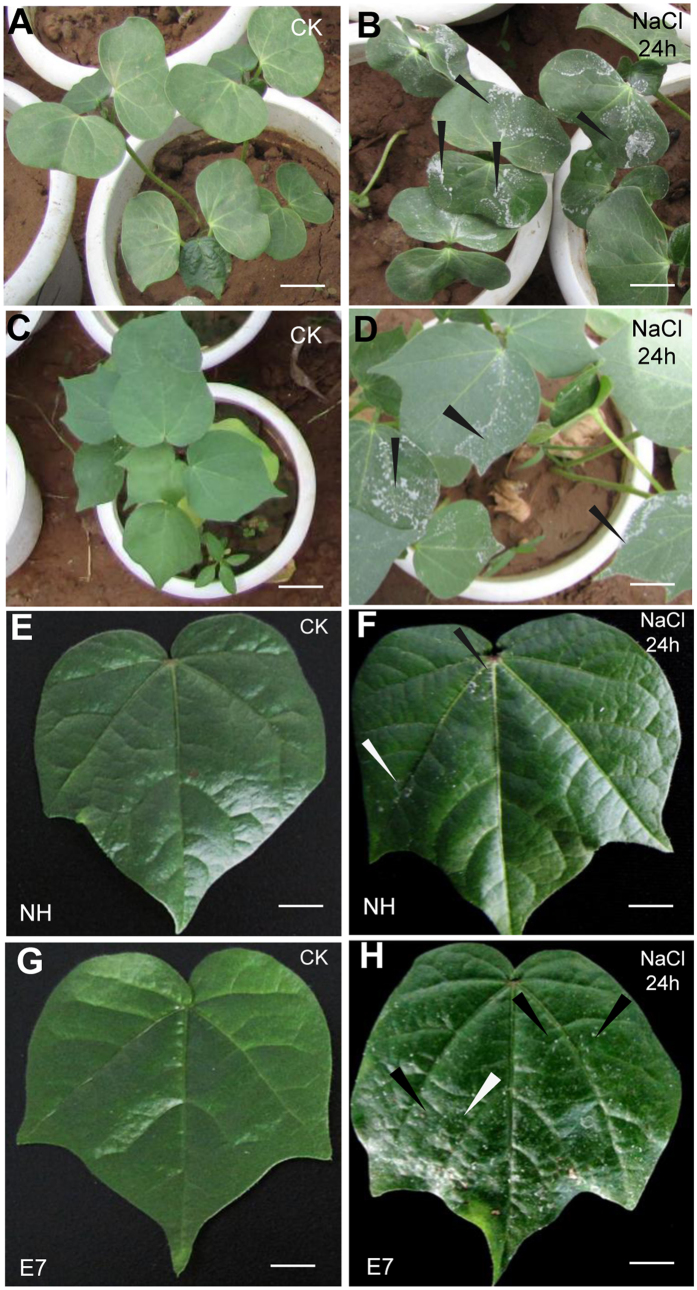
Visual appearance of upland cotton seedlings after salt shock. Abundant salt crystals observed on the adaxial leaf surface of the E7 genotype at the cotyledon stage (**A,B**) and the seeding stage (**C,D**) after treatment with 4% NaCl solution for 24 h. (**E–H**) Salt crystals observed on the NH and E7 adaxial leaf surfaces after 24 h with and without 200 mM NaCl treatment in Hoagland’s solution. Bars = 0.5 cm (**E–H**), 1 cm (**B,D**), and 2 cm (**A,C**). Arrows indicate salt crystals.

**Figure 2:**
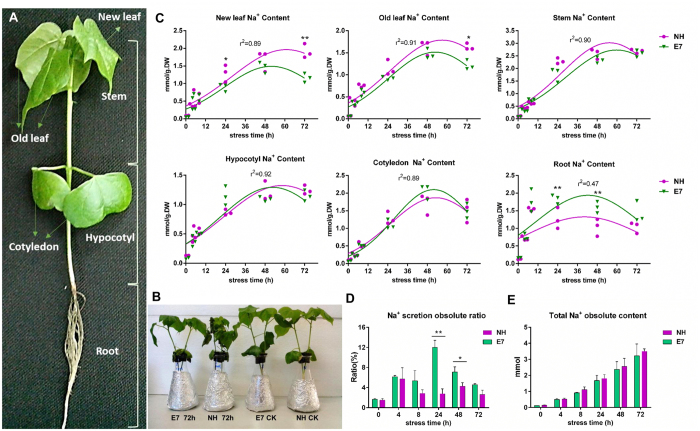
Na^+^ content in the NH and E7 genotypes after NaCl shock at different time points and in different tissues. (**A**) Cotton seedlings were divided into 6 different tissue types and used to measure the relative content of Na^+^ and K^+^. (**B**) Visual appearance of NH and E7 seedlings after 72 h with and without 200 mM NaCl treatment. (**C**) Accumulation of Na^+^ in six NH and E7 tissue types with 200 mM NaCl shock over 72 h. Each symbol represents one biological repeat. (**D**) The percentage of Na^+^ secreted as a proportion of the total Na^+^ content in the whole plant between the two genotypes. The results are the means ± standard errors of three biological replicates. (**E**) The total Na^+^ content in the whole plant between the two genotypes. Single (*) and double (**) asterisks indicate significant differences (P < 0.05, one-way ANOVA).

Furthermore, salt crystals were secreted from the seedling surface after treatment, and the two genotypes appeared to show different amounts of salt secretion (Fig. 1E–H and Supplementary Fig. S1). Therefore, we used the “leaf-washing method” to elute the secretions from the leaf surfaces (of both cotyledons and true leaves) of all 12 samples to quantify the amount of secreted salt ions (Na^+^ and K^+^) after salt treatment. The total Na^+^ content that was secreted was consistently higher in E7 than in NH (10.7% vs. 2.7%, respectively), accounting for the complete absorption of the total Na^+^ content in E7 and NH at the 24-h time point (Fig. 2D). Salinity stress decreased the K^+^ concentration in different tissues at 6 time points compared with that in the control, although E7 was able to maintain slightly higher leaf K^+^ contents than could NH with the 200 mM NaCl shock. The accumulations of K^+^ in the roots, hypocotyls, cotyledons, and stems were similarly affected by salinity stress, whereas no differences between genotypes were observed (Supplementary Fig. S2). However, the related K^+^ content and the percentage of the absolute K^+^ content that was secreted from the leaf surface were ten-fold lower than the corresponding values for Na^+^ at each time point (Supplementary Fig. S3). Considering that the total ingestion of Na^+^ (*in vivo* and secreted) was not significantly different between the two genotypes at any time point (Fig. 2E), we speculated that the Na^+^ secretion capacity might reflect the salt tolerance between the two genotypes.

### Ion distributions in the different cell types of roots and leaves in response to salt shock

**Figure 3:**
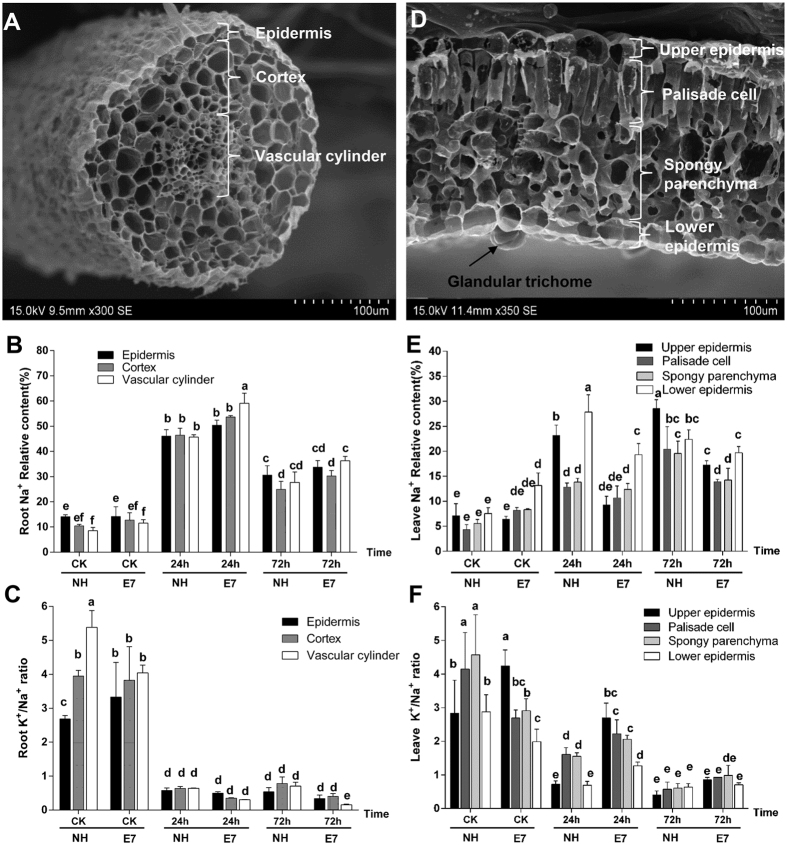
Na^+^ content in NH and E7 after NaCl shock at different time points and in different cells of the roots and leaves. Scanning electron micrographs of the three root cell types with a map scan from the X-ray microanalysis (**A**). The percentages of Na^+^ (**B**) and the K^+^/Na^+^ ratios (**C**) in the epidermis, cortex, and vascular cylinder sections of the roots of *G. hirsutum* seedlings exposed to 200 mM NaCl for 72 h. (**D**) Scanning electron micrographs of the four leaf cell types with a map scan from the X-ray microanalysis. The percentages of Na^+^ (**E**) and the K^+^/Na^+^ ratios (**F**) in the upper epidermis, palisade cell, spongy parenchyma, and lower epidermis sections of leaves of *G. hirsutum* seedlings exposed to 200 mM NaCl for 72 h. The data are the means of 5–8 measurements. The same letter on the bar diagram represents no significant difference (P = 0.05), as determined by the one-way ANOVA. X-ray microanalysis was used to detect the ratios of elements in the roots and leaves. The results were expressed as the percentages of the atomic numbers for particular elements (Na^+^ or K^+^) in the total atomic number for all of the elements (Na^+^, K^+^, Ca^2+^, and Cl^−^) measured in a given region.

The ion contents in the upper and lower epidermal cells and in the cortex and vascular cylinder cells of the leaves and roots were further investigated by X-ray microanalysis under salt stress (Fig. 3A,D). There were no differences in the Na^+^ contents in the root epidermis and cortex between the two genotypes (E7 and NH) with 24 and 72 h of salt treatment; only the vascular cylinder cells showed a significantly increased Na^+^ content in E7 (~1.3 fold) at 24 h (Fig. 3B). There was no difference in the K^+^/Na^+^ ratio in the roots between the two genotypes after stress (Fig. 3C). This result indicated a relatively stable change in the roots between the two genotypes under salt stress.

In the leaves, higher Na^+^ contents were observed in the upper and lower epidermal cells, palisade cells and spongy parenchymal cells of the sensitive line (NH) compared with those of the tolerant line (E7) (Fig. 3E). As a consequence of the decreased Na^+^ and increased K^+^ contents under NaCl treatment, salt-tolerant genotype E7 exhibited much higher K^+^/Na^+^ ratios in the upper and lower epidermal cells, palisade cells and spongy parenchymal cells, especially in the leaves after 24 h of NaCl shock, compared with those of the sensitive type (NH) (Fig. 3F). Based on the above results, we conclude that in the leaves, Na^+^ compartmentation preferentially accumulated in epidermal cells, avoiding excess accumulation in mesophyll cells (palisade cells and spongy parenchymal cells), and less Na^+^ accumulated in the leaf cells (including the epidermal cells and mesophyll cells) of the salt-tolerant genotype than in those of the salt-sensitive genotype. Moreover, the K^+^/Na^+^ ratio was higher in the leaf cells of the salt-tolerant genotype than in those of the sensitive genotype. Considering the Na^+^ content distribution at the tissue and cellular levels, we found that the salt-tolerant genotype (E7) retained significantly less Na^+^ *in vivo* and secreted more Na^+^ than did the salt-sensitive genotype.

### Cotton glandular trichomes on leaves can secrete excess salt

**Figure 4:**
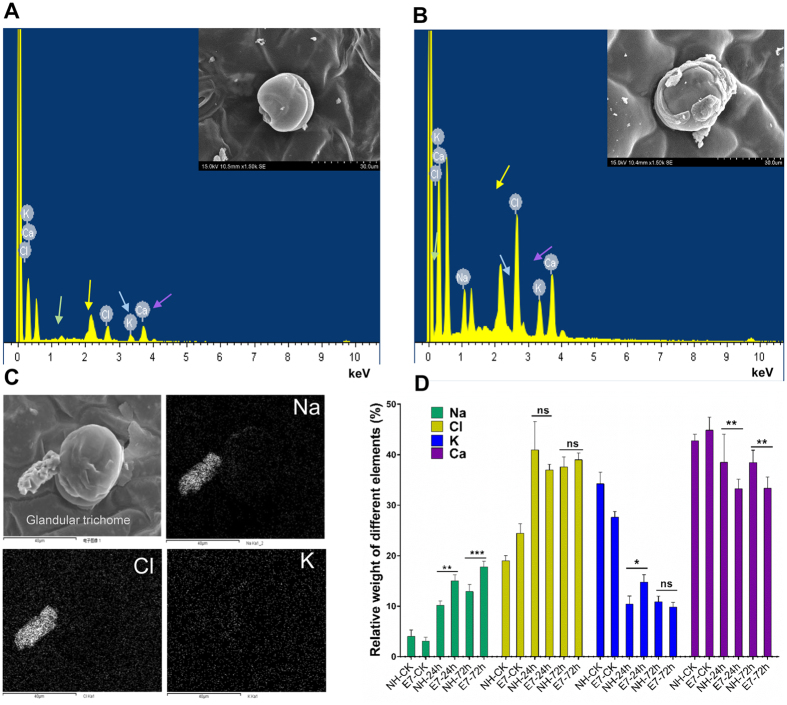
Glandular trichomes respond to NaCl shock. Energy-dispersive X-ray spectroscopy (**A**: ck; **B**: 24 h) and map (**C**: 72 h) showing secreted salt crystals (*) on GTs (**C**) consisting mainly of Na and Cl. Identical highlighted sites on the Na and Cl maps indicate that the two elements coincide with the salt crystals found on the adaxial leaf surface. (**D**) Higher Na, Cl, K, and Ca contents in the secretions around the GTs were detected with greater NaCl shock. The results are the means ± standard errors of five biological replicates. *P < 0.05; **P < 0.01; ***P < 0.001; ns indicates no significant difference in the four elements between NH and E7 genotypes at different time points.

To determine which exact tissue and cell types on the leaf surface have secretory functions in response to salinity shock, secreted Na^+^ ions were detected by SEM and X-ray microanalysis on the adaxial leaf surfaces of NH and E7 after 24 and 72 h of NaCl treatment. First, we confirmed that the salt-secreting structure of the leaves is the glandular trichome (GT); this identification was based on the morphological characteristics of GTs as observed under electron microscopy (Figs 4 and S4) in combination with reports on the trichomes on the leaves of upland cotton^33^,^34^. We also counted the number of GTs on the leaf surface by electron microscopy and found that the number of GTs (per mm^2^) did not differ between the two lines (Supplementary Fig. S4). Second, X-ray microanalysis confirmed that the salt crystals were mainly composed of Na^+^, Cl^−^, K^+^, and Ca^2+^ and were enriched around GTs (Fig. 4) rather than around stoma (Supplementary Fig. S5a,b). Significantly higher concentrations of Na^+^ and Cl^–^in E7 were also observed in the salt crystals of GTs after salt shock compared with those concentrations in the NH genotype (Fig. 4D, and Supplementary Fig. S6). The Na^+^ and K^+^ contents in the secretions on the leaf surface in the salt-tolerant genotype were significantly lower than those in the salt-sensitive genotype, although the Ca^2+^ content showed the opposite trend (Fig. 4D). Taken together, these results suggested that cotton GTs can secrete salt and that the secretion capacity of the salt-tolerant genotype was better than that of the salt-sensitive genotype.

### Na^+^ and H^+^ fluxes in mesophyll cells induced by salt shock

**Figure 5:**
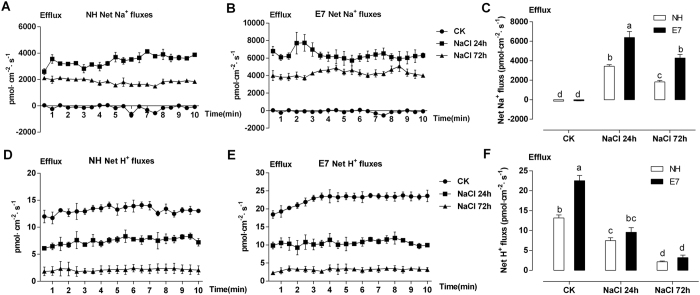
Net Na^+^ and H^+^ fluxes in mesophyll cells of the second true leaves. NH (**A,D**) and E7 (**B,E**) fluxes under 0 and 200 mM NaCl shock for 24 and 72 h. The Na^+^ and H^+^ fluxes were measured immediately after the mesophyll cells were sampled. Each point is the mean of 4–6 individual plants. The bars represent the standard errors of the mean. Panels C and F show the mean flux rates of Na^+^ and H^+^ from 0 to 10 min after the onset of salt shock, which lasted for 24 and 72 h, respectively. Columns labelled with different letters indicate significant differences at P < 0.05.

To further confirm the above result that less Na^+^ accumulated in the mesophyll cells of the salt-tolerant genotype than in those of the salt-sensitive genotype, the stable and constant Na^+^ and H^+^ effluxes in the mesophyll cells were measured using NMT after treatment with 200 mM NaCl for 24 and 72 h (Fig. 5).

NMT revealed that the net Na^+^ flux increased after NaCl shock and that the average Na^+^ fluxes in mesophyll cells of the salt-tolerant E7 genotype were significantly higher (2-fold, P < 0.05) than those of the salt-sensitive NH genotype at 24 and 72 h (Fig. 5A,B). However, the net Na^+^ efflux at 72 h decreased by 30% compared with that at 24 h (Fig. 5C). The net H^+^ efflux in mesophyll cells decreased dramatically at 24 and 72 h in both genotypes; that of the tolerant genotype (E7) decreased more (57.8%) than did that of the sensitive genotype (NH) (43.2%) (Fig. 5D–F). From these results, we hypothesized that the decreased H^+^ efflux rate was caused by the plasma membrane Na^+^/H^+^ antiporter’s high-efficiency pumping of Na^+^ out of the cell and H^+^ into the cell, offsetting the pump H^+^ flux out of the cell.

**Figure 6:**
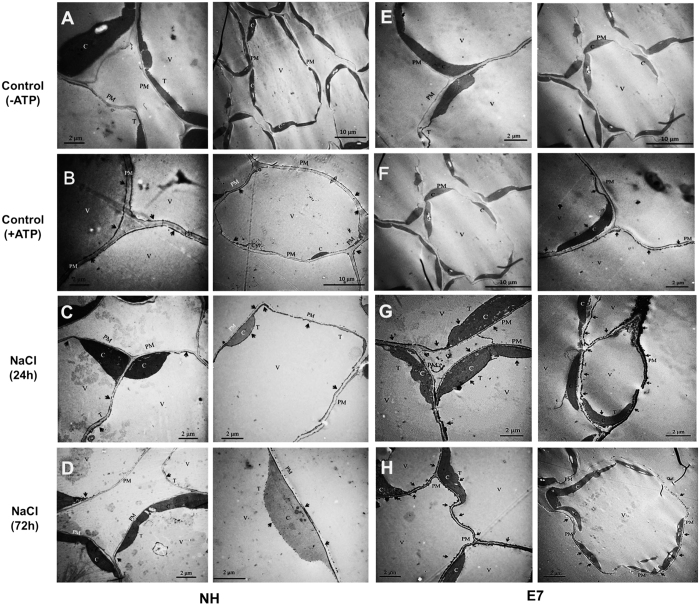
Cytochemical analysis of the H^+^-ATPase activity in leaf cells of E7 (salt tolerant) and NH (salt sensitive) cotton. The staining intensity of the black spot represents the hydrolysis activity of H^+^-ATPase. Blank control (**A,E**) (ATP was absent from the reaction solution); Control (**B,F**); 200 mM NaCl (24 h) (**C,G**); 200 mM NaCl (72 h) (**D,H**). V, vacuole; N, nucleus; C, chloroplast; T, tonoplast; PM, plasma membrane; Cyt, cytosol. Arrows indicate the reaction product of Pb_3_(PO_4_)_2_ precipitates.

We next performed a cytochemical analysis to determine whether the activity of the plasma membrane H^+^-ATPase induced by salt shock could pump protons and maintain electrochemical H^+^ gradients, promoting the secondary active Na^+^/H^+^ antiporter to export more Na^+^ out of the cell. As shown in Fig. 6, the black staining (Pb_3_(PO_4_)_2_) intensity represents the hydrolysis activity of the H^+^-ATPase. The staining intensity was lower because ATP was not added to the incubating solution (as the negative control, Fig. 6A,E). The black staining was clearly seen in control samples of the two genotypes with H^+^-ATPase addition (Fig. 6B,F). The cytochemical results showed that the H^+^-ATPase activity levels in the plasma membrane (PM) and tonoplasts increased 24 or 72 h after salt shock compared with those of the control in the E7 genotype, although the same trend was not observed in the NH genotype at 72 h (Fig. 6C,D,G,H). These results indicated differences in the activity of H^+^-ATPase between the two genotypes, especially after 72 h of salt stress.

### Comparative expression of the candidate genes involved in membrane-associated ion transport and pH homeostasis

**Figure 7:**
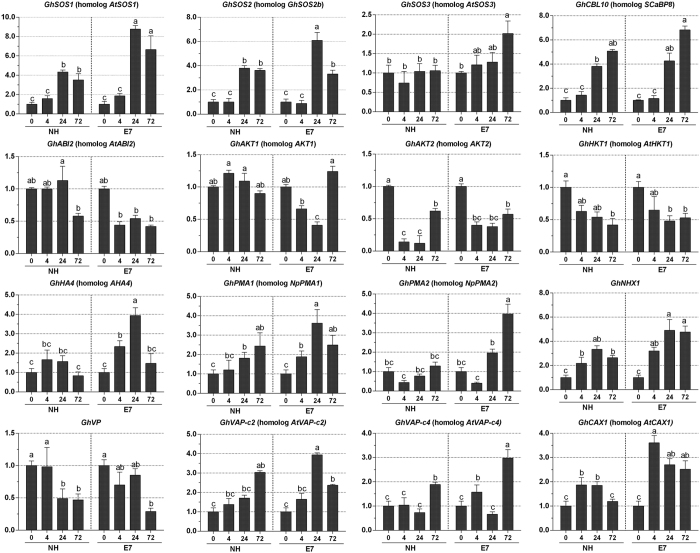
Expression patterns of genes related to salt tolerance in cotton. Gene expression of NH and E7 leaves was compared by qRT-PCR after 0, 4, 24 and 72 h of treatment with NaCl shock. The error bars indicate standard deviations from nine repeats (three biological × three technical repeats). Selected genes encoding pH regulators (PM- and V-ATPase), SOS pathway-related proteins (SOS1, SOS2, SOS3, and ABI2), K^+^ transporters (HKT1 and AKT1), and membrane trafficking-related proteins (NHX1) are shown. capital letters indicate significant differences at the 0.05 level.

To characterize the relationship between the membrane ion transport proteins and upland cotton salt tolerance, the molecular genetic bases of Na^+^ compartmentation, pH homeostasis and aquaporins between E7 and NH leaves were compared using qRT-PCR (Supplementary Table S1). Among the plasma membrane-related ion transporters in cotton leaves, the expression of *GhSOS1, GhSOS2* and *GhCBL10* (SOS3-like calcium-binding protein 8), encoding the salt overly sensitive (SOS) pathway proteins, was significantly up-regulated in the salt-tolerant genotype (E7) after 24 and/or 72 h compared with their expression in the sensitive genotype (NH). *GhAKT1* expression decreased in E7 after 4 and 24 h of treatment and was unchanged in the salt-sensitive NH genotype. *GhAKT2* expression decreased in both E7 and NH under salt shock, with a lesser decrease in E7 and a greater decrease (nearly undetectable) in NH with 24 h of salt shock. The expression of the other K^+^ transporter gene, *GhHKT1,* decreased in both E7 and NH under salt shock. The vacuole membrane-related ion transporters *GhNHX1* and *GhCAX1*, encoding Na^+^ and Ca^2+^ transporters, respectively, were more highly expressed in E7 than in NH at the three time points (Fig. 7).

Among the pH-related transporters, the expression levels of *GhHA4, GhPMA1* and *GhPMA2*, each encoding plasma membrane proton ATPases, significantly increased in E7 compared with those in NH, especially those of the former two genes after 24 h of salt treatment. In E7, the homologous vacuolar proton ATPase subunit *GhVAP-c2* was more highly expressed after 24 h (the difference was significant) and *GhVAP-c4* was more highly expressed after 72 h in E7 compared with in NH (Fig. 7). A comparative analysis of the expression patterns of ion (including proton) transporters and pH homeostasis-related candidate genes supported ion (Na^+^, K^+^ and Ca^2+^) homeostasis, vacuolar membrane trafficking, and the mediation of the tissue tolerance of leaves in response to short-term salt shock.

## Discussion

### Salt secretion from leaf glandular trichomes (GTs) is a new mechanism of Na^+^ compartmentalization in upland cotton

Some halophytes have evolved specific salt glands (“salt hairs”) on the leaf surface for salt secretion. The capacity for secreting salts (by salt glands) is highly correlated with salt tolerance in halophytes^11^. Upland cotton (*Gossypium hirsutum* L.) is the second most salt-tolerant herbaceous crop, although tolerance variation exists among genotypes^20^. The salt secretion phenomenon of the cotton leaf was accidentally identified a half-century ago when researchers tested leaf water potential^35^,^36^. However, few subsequent reports have studied this unique characteristic of cotton. In our study, we directly observed salt secretion on cotton leaves (Fig. 1B,D and Supplementary Fig. S1) and demonstrated that the critical secretion tissues were the GTs, which secreted much more salt than did the stomata following short-term salt shock (Supplementary Figs S5 and S6). Moreover, in the salt-tolerant genotype (E7), the secreted Na^+^ accounted for ~12% of the total Na^+^ content and was much higher than in the sensitive genotype (NH) (~3%) (Fig. 2D). Therefore, we suggested that salt exclusion from leaf GTs is an important mechanism of Na^+^ compartmentalization in cotton. GTs (referred to as glands) on the cotton plant surface secrete various substances, such as high concentrations of cations and lipids, among others^32^, which have protective functions in plant defence^37^. Our study discovered a similar function for the GTs of cotton as that for the salt glands in halophytes. Cotton is not the only glycophyte with salt secretion tissues; similar tissues have been identified in various species, such as microhairs in wild rice^27^, cellular glands in grass^26^, glandular hairs in maize^30^ and hydathodes in *Ficus formosana*^31^.

The different species of the *Gossypium* genus are dispersed throughout different continents^38^. Clearly, this dispersion mainly depended on seawater. Moreover, for cultivated tetraploid cotton (upland cotton), the majority of its wild relatives and geographic landraces were distributed on islands or the coast and exhibited excellent salt tolerance; the capacity for salt secretion in cotton might be a legacy of its ancestors that were grown near the coast and were exposed to long-term high salinity. This hypothesis might also explain why cotton is one of the most salt-tolerant crops.

### The high efficiency of Na^+^ compartmentalization in leaves is a key factor for the salt-tolerant genotype in upland cotton

Ion compartmentalization is one of the most important strategies for avoiding fatal damage to a plant. Different plants present different Na^+^ compartmentalization characteristics. For instance, some halophytes accumulate higher shoot Na^+^ concentrations than do less salt-tolerant halophytes or glycophytes^6^. The priorities of accumulating Na^+^ or Cl^−^ are considered major factors for salt sensitivity^39^. In cotton under salt stress, the main active infector is the cation (Na^+^) rather than the anion (Cl^−^)^22^. Our study confirmed the previous description^22^,^23^ that the Na^+^ content in the shoots (including the stems, true leaves, and cotyledons) was higher than that in the roots under salinity stress (Fig. 2C). In addition, for genotypes with different salt tolerance capacities, the Na^+^ content in leaves of the sensitive genotype (NH) was greater than that of the salt-tolerant genotype (E7) after 24 and/or 72 h. These results were contradictory to the results of Leidi and Saiz^24^ and Ashraf and Ahmad^40^, who reported no differences in the leaf and root Na^+^ content between salt-tolerant and salt-sensitive upland cotton genotype. Meanwhile, we determined that the salt-tolerant E7 genotype could maintain the Na^+^ content in the roots much better than could the salt-sensitive NH genotype, which is consistent with previous reports^41^,^42^. One potential explanation for these differences is that the studies were affected by different salt treatments and genotypes. Interestingly, except for new leaves, the roots also showed a significant difference in the Na^+^ content. More Na^+^ was detected in the salt-tolerant genotype (E7) than in the sensitive genotype (NH) (Fig. 1F,H). We further confirmed that a high level of Na^+^ was mainly concentrated in the root vascular cylinder cells at the cellular level (Fig. 3B). This finding was different from reports on wild-type Arabidopsis^43^ and citrus^44^, where a significant amount of Na^+^ was retained in the epidermal and cortical cells. We might reasonably conclude that in cotton, although the roots of the salt-tolerant genotype (E7) had the capacity to retain Na^+^, the effect was temporary (the Na^+^ content in the roots began to decrease after 48 h of salt treatment, while the Na^+^ content continuously increased in new leaves during this period) and limited (the Na^+^ content in the roots accounted for only a small proportion of the total Na^+^ content in the whole plant), most of the Na^+^ from the roots was finally transported to the shoots (Fig. 2C). The Na^+^ compartmentalization capacity of the shoots played a more important role in cotton.

We found that different types of leaf cells exhibited discriminatory compartmentation capacities between the two genotypes, and Na^+^ accumulated in epidermal cells more than in mesophyll cells, especially in the sensitive genotype (NH) (Fig. 3E). Additionally, the salt-sensitive genotype (NH) exhibited a greater Na^+^ accumulation in the leaves than did the salt-tolerant genotype (E7) (after 24 and 72 h of NaCl shock), which was consistent with the corresponding tissue level (Fig. 2C). These results were caused by more Na^+^ being secreted through GTs in the salt-tolerant genotype than in the sensitive genotype (Fig. 2D). Furthermore, this increased accumulation was likely caused by the strongly selective distribution of Na^+^ and K^+^, with Na^+^ being pumped out of and K^+^ being transported into the mesophyll cells in the salt-tolerant genotype (E7) under salt shock (Fig. 3E,F). The optimal cytosolic K^+^/Na^+^ ratio could be maintained by either restricting Na^+^ accumulation in plant tissues or cells (via the Na^+^/H^+^ transporter) or preventing K^+^ loss from cells (via K^+^ channels). K^+^ channels include both low-affinity [e.g., non-selective cation channels (NSCC) and inward-rectifying channels (AKT, KAT)] and high-affinity (e.g., HKT) transporters^45^. In our study, the changes in *GhAKT1* expression demonstrate that the K^+^ influx will decrease in E7 but not in NH. Similar results have also been reported in rice by Golldack *et al*.^46^. *GhAKT2* expression suggested that the active uptake of K^+^ was restrained by the hyperaccumulation of Na^+^ but did not differ between the E7 and NH genotypes. The AKT2/3 K^+^ channel is involved in the recirculation of K^+^ throughout the phloem under salinity^47^ and transporting K^+^ into leaf mesophyll cells^48^. These results suggest that the hyperaccumulation of Na^+^ would inhibit the activity of the AKT channel, leading to intracellular Na^+^ ion blockage while avoiding K^+^ leakage. The salt-tolerant genotype had a greater ability to maintain high K^+^/Na^+^ ratios in mesophyll cells (Fig. 3F). HKT-type transporters are another possible pathway for Na^+^ influx. In Arabidopsis, knocking out the *AtHKT1* gene leads to hypersensitivity to salinity with more Na^+^ in the leaves because Na^+^ could be sourced from the xylem while directly stimulating K^+^ loading by AtHKT^49^. In our study, *GhHKT1* (homologous *AtHKT1*) expression decreased in both E7 and NH under salt shock and was lower in E7 than in NH. These results indicated that the salt-tolerant genotype could reduce the inhibition of K^+^ uptake due to the high Na^+^ concentration and low-affinity Na^+^ absorption, thereby reducing the Na^+^/K^+^ ratio in the leaves. Kader *et al*. reported that the down-regulation of *OsHKT1* in leaf mesophyll cells in the salt-tolerant line was caused by hindering Na^+^ transport into these metabolically active cells^50^. Unfortunately, the difference in *GhHKT1* expression between the two genotypes was not significant (Fig. 7). Taken together, given the above-described salt-dependent expression regulation, *GhAKT1* expression indicated the involvement of inward-rectifying channels in the regulation of the K^+^/Na^+^ ratio in leaves under salt stress.

**Figure 8:**
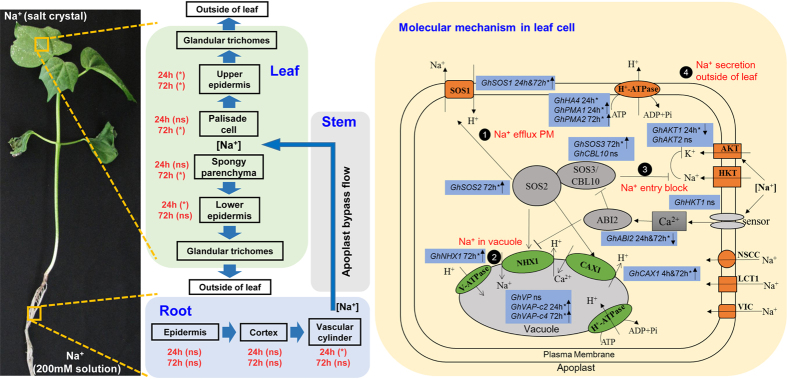
Distribution and transportation of Na^+^ in *G. hirsutum*. Schematic diagram presenting the Na^+^ distribution and transportation in cotton at the tissue and cellular levels from the soil through the roots and up to the shoots. The molecular mechanism of intracellular Na^+^ compartmentalization is also presented. For roots and leaves, *indicates that the Na^+^ content was significantly different between the two species at various time points. Regarding the molecular mechanisms in leaf cells, (1) Na^+^ was extruded or effluxed from the cytosol of photosynthetic cells by PM H^+^-ATPase and SOS1. (2) Na^+^ was efficiently sequestered into vacuoles; this step was possibly mediated by a tonoplast proton pump and NHX antiporters. (3) The influxed Na^+^ in leaf cells was blocked by the HKT1 transporter. (4) Na^+^ was secreted from the leaves by glandular trichomes. Blue boxes represent Na^+^ transportation-related genes between NH and E7 after 24 and/or 72 h; *indicates significantly different expression; ns indicates not significant; upward-pointing arrows indicate genes that were up-regulated in the salt-tolerant genotype; downward-pointing arrows indicate genes that were down-regulated.

Furthermore, we confirmed that active Na^+^ and H^+^ extrusion in mesophyll cells of the salt-tolerant genotype (E7) contributed to Na^+^ homeostasis under salt shock (Figs 5 and 6). Such a net Na^+^ efflux is commonly observed in other species, such as bean leaf mesophyll^51^ and populus^52^, wheat^53^ and pepper^54^ roots after NaCl treatment. Under salt stress conditions, Kong *et al*.^55^ found that the cotton root net Na^+^ efflux was positively correlated with the net H^+^ influx as a result of an active Na^+^/H^+^ antiporter across PM H^+^-ATPase. Unlike previous reports, our work presented both net Na^+^ and H^+^ in the mesophyll cells exhibiting efflux (Fig. 5). Two major transmembrane proteins manage Na^+^ extrusion from the cytosol: the Na^+^/H^+^ antiporter is energy dependent and driven by the electrochemical gradient created by PM H^+^-ATPase^56^. Our result might be due to the strong activity of PM H^+^-ATPase (Figs 6 and 7). The decreased H^+^ efflux corresponding to the Na^+^ efflux suggested that the Na^+^ extrusion in salt-stressed cotton leaves was mainly attributed to an active Na^+^/H^+^ antiporter across the high-activity PM H^+^-ATPase. This salt shock increased the *GhSOS2, GhSOS1, GhSOS3, GhPMA1*, and *GhPMA2* expression levels in the leaves, and these levels were different between the two genotypes (Figs 7 and 8). Salt stress increases the SOS1 Na^+^/H^+^ antiporter in the SOS pathway and facilitates Na^+^ efflux across the plasma membrane, regulating H^+^-ATPase activity^1^,^5^.

The Na^+^ compartment in the vacuole is also important for osmotic adjustment because it decreases the Na^+^ concentration in the cytosol^57^. Na^+^ sequestration into vacuoles occurs via the expression and activity of Na^+^/H^+^ antiporters^58^,^59^ and two electronic H^+^ pumps: the vacuolar H^+^-ATPase (V-ATPase) and the vacuolar pyrophosphatase (V-PPase)^60^. The activity of H^+^-ATPase in the vacuolar membrane was also observed and increased in E7 compared with NH after 24 h of salt shock (Fig. 6C,G). The qRT-PCR results indicated that salt shock significantly enhanced *GhNHX1, GhVAP-c2*, and *GhVAP-c4* expression in E7 compared with NH (Figs 7 and 8). These results demonstrated that the salt-tolerant genotype had a greater ability to increase Na^+^ sequestration into the vacuoles and further modulated ion homeostasis to cope with high salinity. According to the observations from the transmission electron microscopy and energy spectrum analysis (TEM-EPA), the Na^+^/H^+^ transporter and H^+^-ATPase in the plasma membrane play key roles in transporting Na^+^ from mesophyll cells, and these transport proteins corresponding to the vacuole membrane also play roles in salt tolerance (Fig. 8). The sequestrated Na^+^ secreted from plants through GTs could further enhance the salt tolerance of cotton. Our findings provide knowledge and a novel perspective to better understand the salt tolerance mechanism in cotton.

## Materials and Methods

### Plant materials, seedling culture conditions and salt treatment

Seeds of two upland cotton (*G. hirsutum*) lines, salt-tolerant Earlistaple 7 (E7) and salt-sensitive Nan Dan Ba Di Da Hua (NH), were obtained from the National Mid-term Genebank of the Institute of Cotton Research, Chinese Academy of Agricultural Sciences (ICR-CAAS)^61^. In a previous physiological study, these two cotton lines showed significant differences in salt tolerance at third-true leaf stages^62^.

Hand-selected seeds were sterilized in ethanol [70% (v/v)] for 15 s and sodium hypochlorite [4% (w/v)] for 15 min. The seeds were submerged in sterile water for 12 h and then sown in sterile silica sand with water in a chamber at 30 °C. After 3 days, uniform seedlings were transplanted to aerated hydroponic containers (600 × 350 × 120 mm) containing half-strength Hoagland’s solution (pH 6.0)^63^ and were grown in a phytotron (Kerui Inc., Zhengzhou, Henan, China) under optimized conditions (28/22 °C day/night, 60–80% relative humidity, and a 14-h photoperiod under 450 μmol·m^−2^·s light intensity). When the seedlings had produced three true leaves (approximately 14–16 days after seedling emergence), they were maintained under NaCl-free conditions before the onset of salt treatment. Uniform seedlings (three true leaf stage) were randomly divided into two groups; one group was carefully placed into tanks filled with 200 mM NaCl half-strength Hoagland’s solution, and the other group was transferred into tanks without NaCl half-strength Hoagland’s solution to serve as the control.

### Measurement of ion accumulation at the tissue level

In this experiment, the ion accumulation in different tissue types from both samples was measured. First, we harvested NH and E7 seedlings that had been exposed to the NaCl solutions for 0, 4, 8, 24, 48, and 72 h (5 plants per treatment in each replicate, with a total of three replicates). Then, the leaf mass (cotyledons and true leaves) of each sample was accurately measured (total 12 samples = 2 cotton lines × 6 time points), and the leaves were then hand-dipped in 40 mL of double-distilled water (room temperature) and shaken gently for approximately 1 minute to easily to dissolve salt crystals and to avoid damaging the leaf tissue. The 12 leaf surface samples were stored in 50-mL centrifuge tubes for further experiments. The roots were also washed with distilled water for approximately 1 minute. Each sample was separated into six tissue types: roots, hypocotyls, cotyledons, stems (including leaf stalks), old leaves, and new leaves (Fig. 2A).

All of the samples were dried at 60 °C until reaching a constant dry weight. Then, the Na^+^ and K^+^ concentrations of the 14 samples (six tissues and the corresponding salt ion solution per sample) were determined via inductively coupled plasma-optical emission spectrometry (ICP-OES) (Optima 2100 DV; Perkin-Elmer, Wellesley, USA) according to the manufacturer’s instructions^64^. Three biological replicates for each control and time-treated sample were performed for this experiment.

### Measurement of the ion accumulation at the cellular level

#### a) Scanning electron microscopy (SEM) and X-ray microanalysis

The mature leaves (the first true leaves) and roots (elongation zone) of E7 and NH were sampled 0, 24 and 72 h after 200 mM NaCl shock treatments. The seedling roots (2–3 mm long) or leaf sections (2 × 2 mm) for each sample were divided into two groups: one for cellular element examination and another for the cytochemical staining of H^+^-ATPase (see part c).

The samples being tested were placed into copper mesh and then immediately into liquid nitrogen for quick freezing. The samples were then transferred into a freeze dryer for 6 d at −110 °C for the roots and 3 d at −60 °C for the leaves; after 24 h, the temperature was returned to room temperature. The samples were then fixed to the sample stage with conductive glue and plated on a layer of gold-plated membrane using a high-vacuum sputter coater, after which the samples were scanned using a Hitachi S-3400N SEM-EDAX (EX-250; Horiba Ltd., Kyoto, Japan).

For each cellular compartment, 5–8 measured regions were scanned, with a counting time of 60 s. The following conditions were used: an SEM accelerating voltage of 10 kV, a sample tilt angle of 35 °C, and an X-ray energy-dispersive spectroscopy quantitative analysis using non-standard procedures for qualitative and relative quantitative analyses^65^. The relative amounts of K^+^ and Na^+^ were expressed as percentages of the total atomic number for all of the major elements (K^+^, Na^+^, Ca^2+^, and Cl^−^) that were detected from the root and leaf sections of each sample. Approximately 5–8 regions on the leaf surface and transverse sections were examined. We also scanned the glandular trichomes and stomata of each leaf sample to determine the ion concentrations in a selected field of vision and used the counting method as described above.

#### b) Measurements of the net Na^+^ and H^+^ fluxes using NMT

The net Na^+^ fluxes were measured at the Younger USA (Xuyue, Beijing) NMT Service Center using Non-invasive Micro-test Technology (NMT 100 Series, Younger USA LLC, Amherst, USA) and iFluxes/imFluxes1.0 software (Younger USA, LLC, Amherst, USA) as previously described^52^. First, the mesophyll cells of the second true leaves were exposed after the leaves were peeled off the epidermal layer. Then, the Na^+^ and H^+^ concentration gradients were measured from the prepared mesophyll cells by moving the electrode repeatedly between two positions adjacent to the mesophyll cell in a pre-established excursion (30 μm) and at a programmable frequency in the range of 0.3–0.5 Hz. The NMT could measure ionic fluxes to the picomolar level, but the measurements had to be slow, at approximately 5 to 6 s per point.

Pre-pulled and silanized glass micropipettes (2- to 4-μM aperture, XY-SJ-Na, XY-SJ-H; Xuyue Sci. & Tech. Co., Ltd) were first filled with a backfilling solution (Na, 250 mM NaCl; H, 40 mM KH_2_PO_4_ and 15 mM NaCl, pH 7.0) to approximately 1.0 cm from the tip. Then, the micropipettes were front-filled with 25-μM columns of selective liquid ion-exchange cocktails (LIXs: Na, Sigma 71178; H, Sigma 95293). An Ag/AgCl wire electrode holder (XY-ER-01; Xuyue Sci. and Tech. Co. Ltd.) was inserted into the back of the electrode to establish electrical contact with the electrolyte solution. YG003-Y05 (Younger USA) was used as the reference electrode. Ion-selective electrodes of the following target ions were calibrated prior to the flux measurements: (i) 0.5 mM NaCl, 0.1 mM KCl, 0.1 mM CaCl_2_, and 0.3 mM MES, pH 5.5; and (ii) 5 mM NaCl, 0.1 mM KCl, 0.1 mM CaCl_2_, and 0.3 mM MES, pH 6.5.

Only electrodes with Nernstian slopes >53 mV/decade were used in the present study. The ion flux was calculated by Fick’s law of diffusion: J = −D_0_ · (dc/dx); where J is the ion flux (unit: picomole·cm^−2^ · s^−1^), dc is its concentration gradient, dx is the distance between the two points (usually 30 μm), and D_0_ is the diffusion constant. The direction of the flux was derived from Fick’s law of diffusion.

After exposure to 200 mM NaCl shock treatments (for 0, 24 and 72 h), E7 and NH leaf segments (the second true leaves) with areas of 5 × 3 cm were quickly sampled for Na^+^ and H^+^ flux measurements after the epidermal cells were peeled off. To decrease the effect of salt release on flux recording, the segments were rinsed with redistilled water and immediately incubated in the measuring solution to equilibrate for 30 min, and the flux rate decreased gradually to a steady level within 10 min. After equilibrating for 30 min, the leaf mesophyll cells were transferred to a measuring chamber containing 10–15 mL of a fresh measuring solution. After the leaves were immobilized on the bottom, ion flux measurements began. The measured areas of the mesophyll cells could be visualized and defined under the NMT microscope. Na^+^ ions were monitored in the following solutions: 0.5 mM NaCl, 0.1 mM KCl, 0.1 mM CaCl_2_, and 0.3 mM MES, pH 6.0 adjusted with choline/NaOH and HCl. The microvolt differences (ΔμV) were then exported as raw data before they were imported and converted into net fluxes using JCal V3.2.1 (a free MS Excel spreadsheet, http://www.youngerusa.com). Each ion (Na^+^ and H^+^) for each sample was measured with five biological replicates.

#### c) Cytochemical analysis of membrane H^+^-ATPase

The histochemical localization of ATPase activity is based on an indicator (insoluble Pb_3_(PO_4_)_2_ precipitate). The staining intensity could represent the hydrolysis activity of the ATPase. To detect the membrane H^+^-ATPase activity, sections were cut from the same leaves that were used for X-ray microanalysis. The black staining (Pb_3_(PO_4_)_2_) intensity was used to indicate the ATPase activity, as described by Ma *et al*.^66^.

### Expression testing of the involved genes by real-time RT-PCR

For gene expression studies, the leaf samples of genotypes E7 and NH were collected for total RNA extraction 0, 4, 24 and 72 h after 200 mM NaCl shock treatments. The qRT-PCR method followed was described by Peng *et al*.^54^. Salt overly sensitive (SOS) pathway- and vacuolar Na^+^/H^+^ antiporter-related candidate genes were selected from the literature^43^. Nucleotide sequences for candidate genes were obtained by BLAST from the BGI *Gossypium hirsutum* (AD)_1_ genome^67^ (ftp://ftp.bioinfo.wsu.edu/species/Gossypium_hirsutum/CGP-BGI_G.hirsutum_AD1genome/genes/). The primers that were used in this study are listed in Supplementary Table S1, and each gene assessment was performed with three biological replicates.

### Statistical analysis

For experiment 1, each data point represented the mean of three biological replicate treatments, with each treatment consisting of at least five plants. The values in the figures and tables are expressed as the means ± SEs. The statistical analyses were performed using Tukey’s two-way analysis of variance (ANOVA) in IBM SPSS Statistics v19.0 (SPSS Inc., Chicago, IL, USA). *P-values* < 0.05 were considered statistically significant. For experiment 2, the X-ray microanalysis data were subjected to ANOVA, and significant differences between the means were determined using Duncan’s multiple-range test. The different small letters between two samples represent significance at *P-value* < 0.05.

### Abbreviations

GT, glandular trichome; E7, Earlistaple 7; NH, Nan Dan Ba Di Da Hua; qRT-PCR, quantitative real-time PCR; SOS, salt overly sensitive; PM, plasma membrane; NMT, non-invasive micro-test technology, SEM, Scanning electron microscopy.

## Additional Information

**How to cite this article**: Peng, Z. *et al*. Na^+^ compartmentalization related to salinity stress tolerance in upland cotton (*Gossypium hirsutum*) seedlings. *Sci. Rep.* **6**, 34548; doi: 10.1038/srep34548 (2016).

## Supplementary Material

Supplementary Information
