# Supplementary material for: Na+ compartmentalization related to salinity stress tolerance in upland cotton (Gossypium hirsutum) seedlings
Source: Sci Rep. 2016 Oct 4;6:34548. doi: 10.1038/srep34548 (PMC5048304; doi:10.1038/srep34548)
Supplement: Supplementary Information [file srep34548-s1.pdf]

Supplementary Data

Na^+ compartmentalization related to salinity stress tolerance in upland cotton (*Gossypium hirsutum*) seedlings

Zhen Peng [1,2,§], Shoupu He[1,§], Junling Sun[1], Zhaoe Pan[1], Wenfang Gong[1], Yanli Lu[2*] and Xiongming Du[1*]

[1]State Key Laboratory of Cotton Biology/Institute of Cotton Research, Chinese Academy of Agricultural Sciences, Anyang, Henan 455000 China

[2] Maize Research Institute of Sichuan Agricultural University/Key Laboratory of Biology and Genetic Improvement of Maize in the Southwest Region, Ministry of Agriculture, Wenjiang, Sichuan 611130 China

§These authors contributed equally to work.

*Correspondence should be addressed to Xiongming Du (dujeffrey8848@hotmail.com) and Yanli Lu (yanli.lu82@hotmail.com)

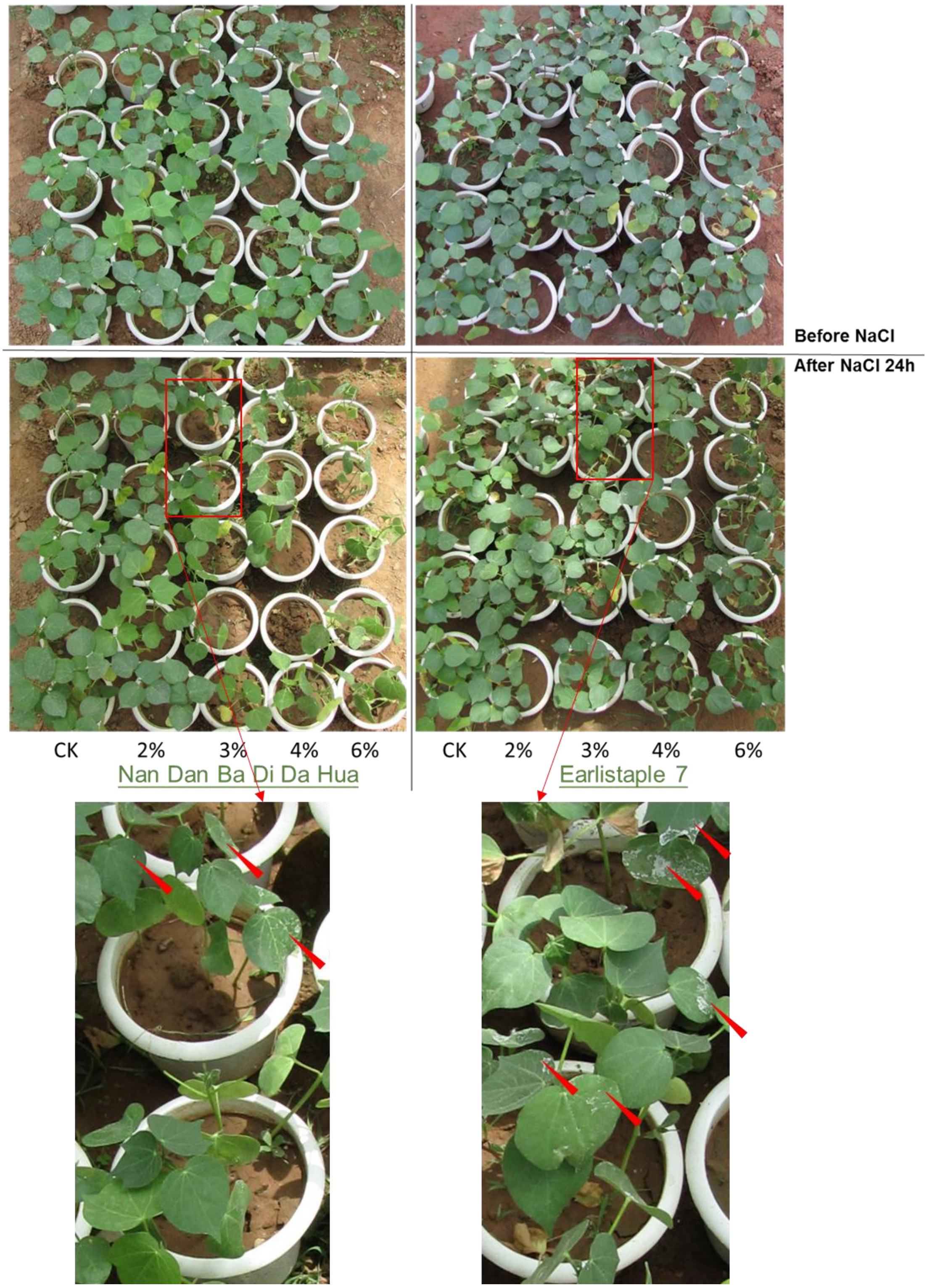

Figure S1. Visual appearances of Nan Dan Ba Di Da Hua (NH) and Earlistaple 7 (E7) leaves before and after 24 h of exposure to NaCl solution shock with different salinity concentrations (2-6%, w/v) in the soil under glasshouse conditions (the temperature was 20-32℃). The red arrows point to salt crystals observed on the adaxial leaf surface.

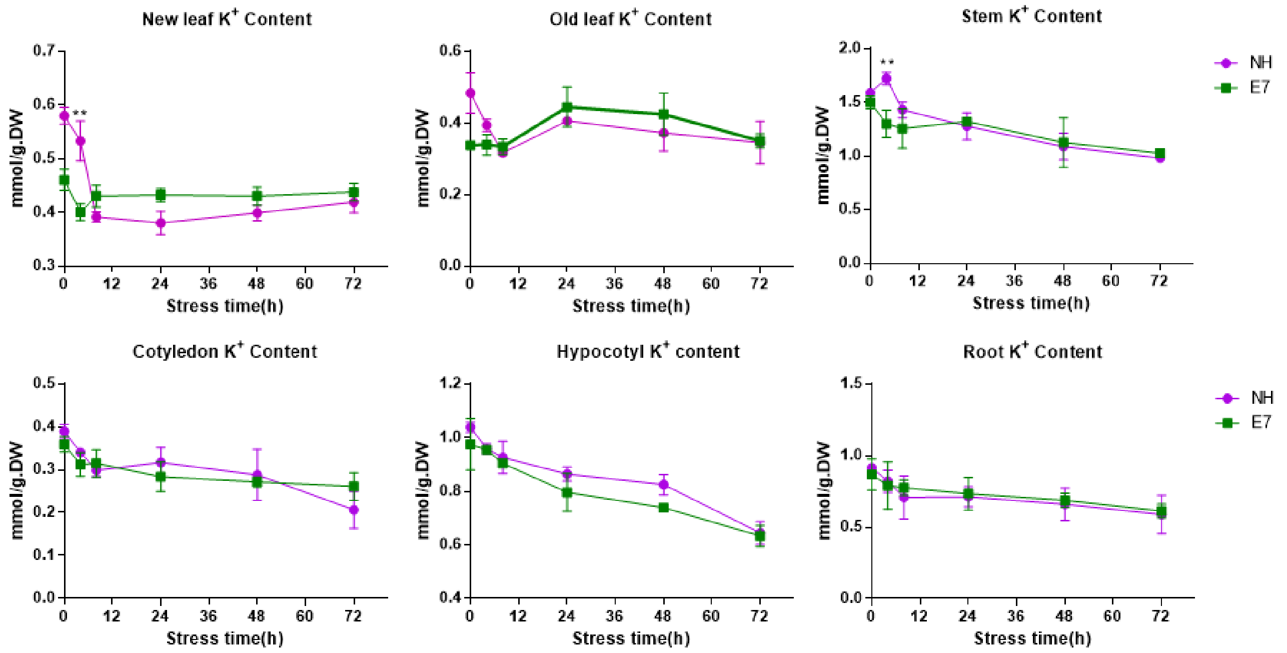

Figure S2. K^+ content (a) in six tissue types of NH and E7 with 200 mM NaCl shock over 72 h. Bars represent the averages of the treatments with or without NaCl (200 mM), means ± standard errors (n = 3). All statistical analyses between genotypes for each time point showed no significant differences except new leaf and stem K^+ content at 4h time point (P-value <0.001).

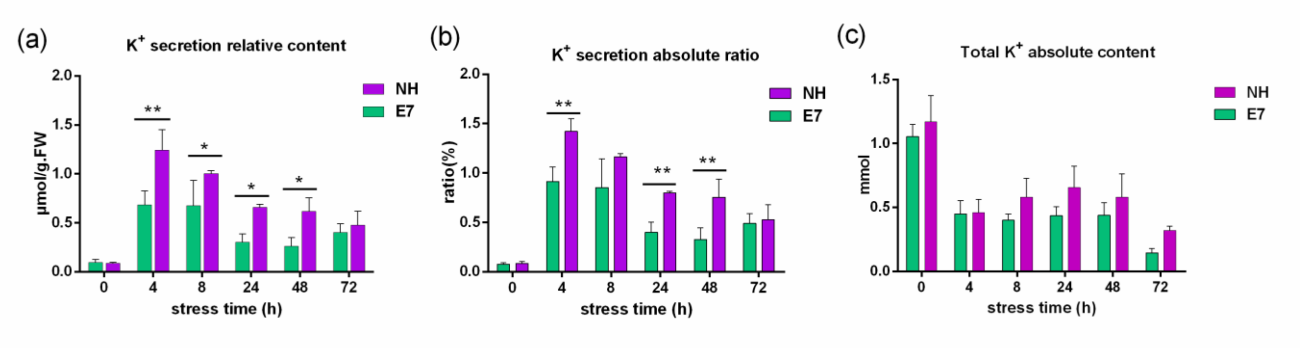

Figure S3. K^+ contents in the secretions were detected from the NH and E7 genotypes after NaCl shock at different time points. (a) The K^+ relative contents in the secretions were detected. The results are the means ± standard errors of three biological replicates. (b) The percentage of K^+ secretion accounts for the whole plant uptake absolute Na^+ content between the two genotypes. (c) Total K^+ absolute content in the whole plant uptake absolute K^+ content between the two genotypes. Single (*) and double (**) asterisks indicate significant differences ($P < 0.05$, one-way ANOVA). The results are the means ± standard errors of three biological replicates.

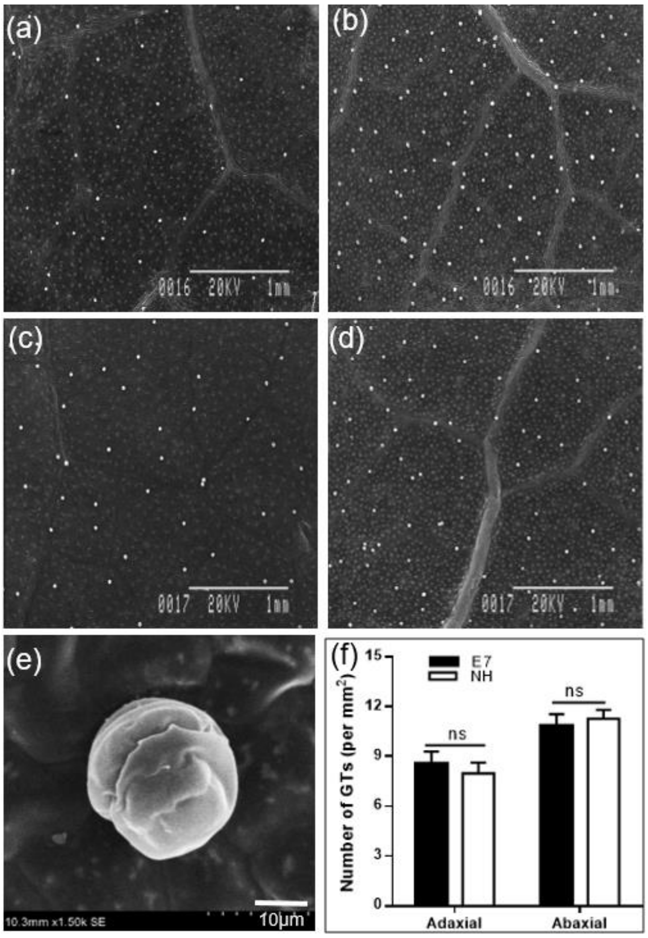

Figure S4: Scanning electron micrographs of upland cotton leaves. the adaxial (a,c) and abaxial (b,d) surfaces of E7 and NH genotype leaves, respectively.The white points means the glandular trichome (GT). (e) Magnified view of a GT. (f) Comparisons of the GTs counts on the adaxial and abaxial surfaces of the leaves of NH and E7. "ns" means no difference between the two genotype. The results are the means ± standard errors of fifty replicates (3 visible area × 5 leaves).

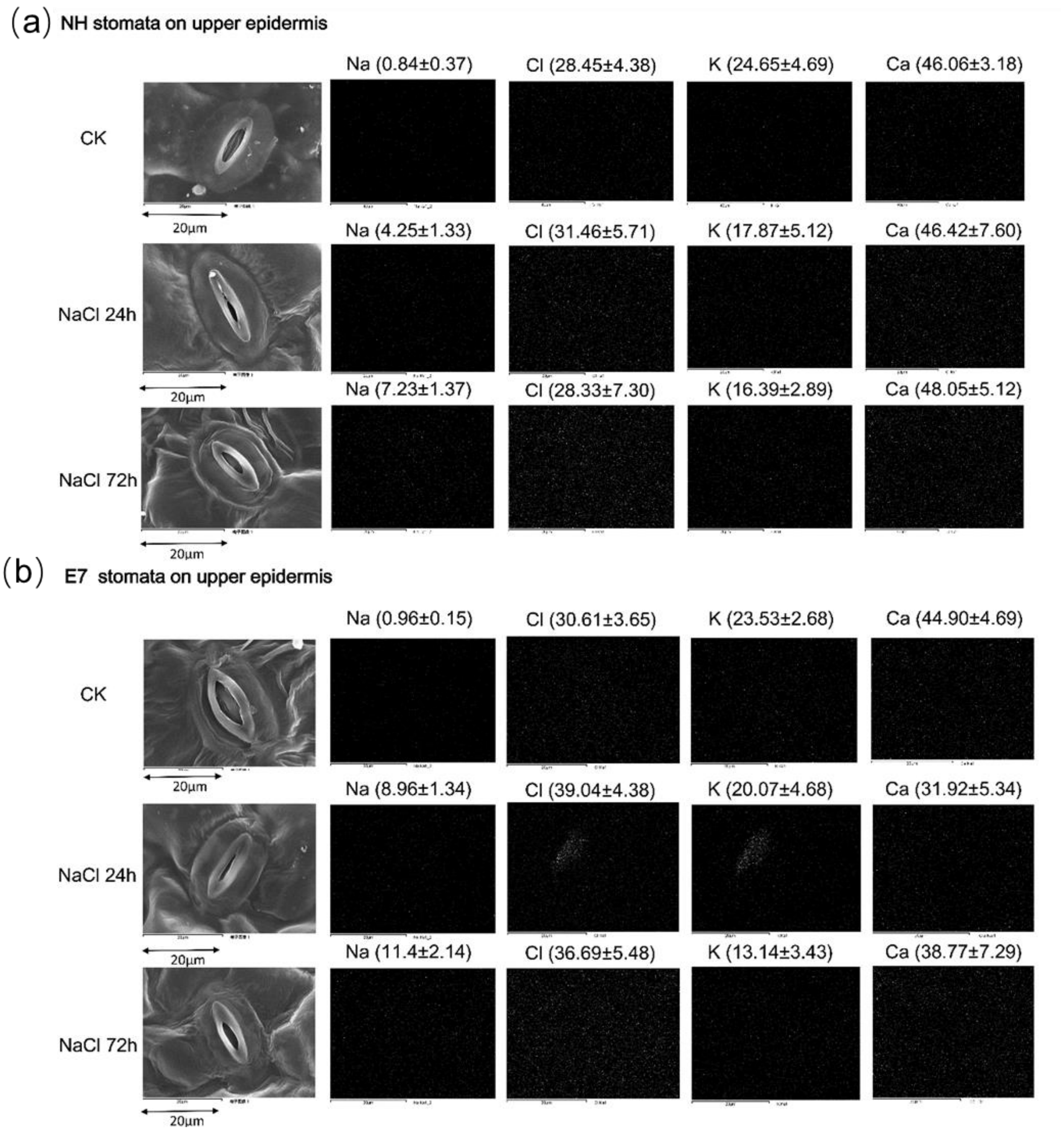

Figure S5. Scanning electron micrographs of the stoma of the adaxial leaf surface with a map scan using X-ray microanalysis. The percentages of Na^+, K^+, Ca^{2+}, and Cl^- around the stoma of the upper epidermis sections of leaves of NH (a) and E7 (b) seedlings in 200 mM NaCl at 0, 24 and 72 h. The data are the means of 5-8 measurements. The results show the percentages of the atomic number for each element in the total atomic number for all of the elements (Na^+, K^+, Ca^{2+}, and Cl^-) measured in a selected region.

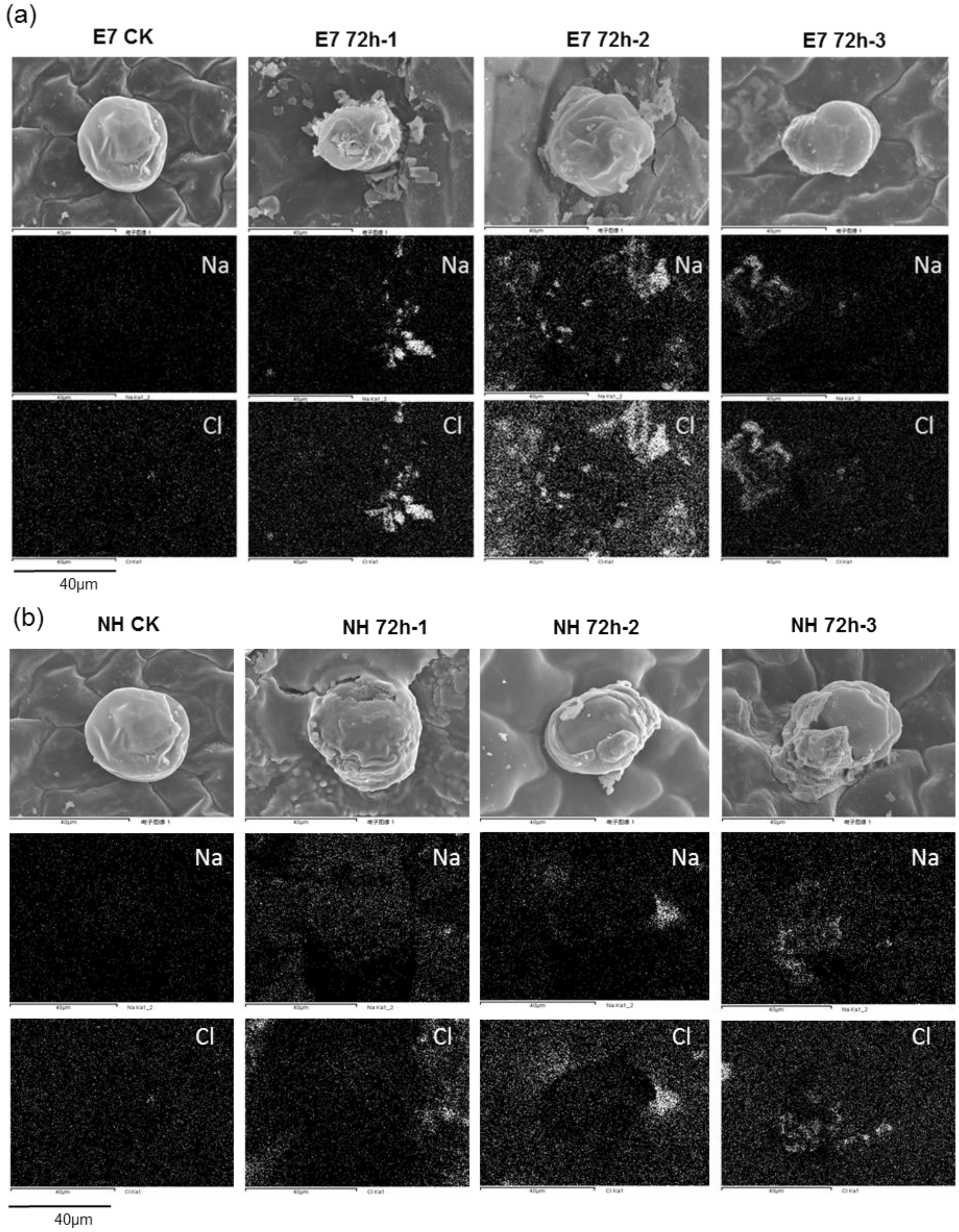

Figure S6. Glandular trichomes (GTs) respond to NaCl shock. Energy dispersive X-ray spectroscopy map (0 and 72 h) showing secreted salt crystals (*) on the GTs of the E7 (a) and Nan Dan Ba Di Da Hua genotype (b).

Table S1: List of primers used to detect genes in the qRT-PCR analyses

Gene ID	Name	Homologous	Annotation		Primer sequence (from 5'to 3')
AY305733	*GhActin*	*GhActin*	Housekeeping gene	F	ATCCTCCGTCTTGACCTTG
				R	TGTCCGTCAGGCAACTCAT
CotAD_65716	*GhNHX1*	*GhNHX1*	Gossypium hirsutum Na/H antiporter (NHX1)	F	GCCAGGACTCTTTTGATGAT
				R	AGTGTGTGCTGGAGTTGTAAG
CotAD_64499	*GhVP*	*GhVP*	Pyrophosphate-energized Vacuolar H(+)- pyrophosphatase	F	TATGGTGATGACTGGGAAGG
				R	GATACAACATGCCCGTGAAG
CotAD_69068	*GhSOS1*	*AtSOS1*	Sodium/hydrogen exchanger 7 (Na(+)/H(+) exchanger 7) (NHE-7) (Protein SOS1)	F	GTGATGGCATTCGACTTTGG
				R	GACCAGCAAGCAGAACCATTT
CotAD_17719	*GhSOS2*	*GhSOS2b*	CBL-interacting serine/threonine-protein kinase 24/CIPK24 (GU188961)	F	GCATGAGGTTCACTGTGGAAG
				R	AAGTCCGACCCCTTGCTGTAG
CotAD_19826	*GhSOS3*	*AtSOS3*	Calcineurin B-like protein 4/CBL4	F	TTCTTGCTGCTGAAACACCT
				R	AACTCCTCATGCTCGATAAA
CotAD_65286	*GhCBL10*	*SCaBP8*	Calcineurin B-like protein 10/SOS3-LIKE	F	CTCGCCGTATGGAGTTTGGA
				R	CGTCTTGCCAATGAATCCCG
CotAD_03278	*GhCAX*	*AtCAX1*	Vacuolar cation/proton exchanger 1 (Ca(2+)/H(+) antiporter CAX1)	F	TGCGTCCTTGTCTCTTGGATT
				R	GGATAGAGCGAGAGAGCCAGTTT
CotAD_23707	*GhABI2*	*AtABI2*	Protein phosphatase 2C 77 (AtPP2C77) (Protein phosphatase 2C ABI2)	F	CAGTTTCGGGGGCAATAGGT
				R	TATCAGGCACGATTGGCAGG
CotAD_17687	*GhAKT1*	*AKT1*	Potassium channel AKT1	F	TTGCATTGCTCAAACACTAGCC
				R	TCCCACGAGCTAGCATGTTTT
CotAD_31740	*GhAKT2*	*AKT2*	Potassium channel AKT2/3	F	ACTCCAAGGGATGGGTCACT
				R	GCGCCGATGAACTGAGAAAC

CotAD_51484	*GhHA4*	*AHA4*	ATPase 4, plasma membrane-type (EC 3.6.3.6) (Proton pump 4)	F	AACCGACCGCAAGGACATAA
				R	ACCAACTCCTTGCTCGTGTT
CotAD_43378	*GhPMA1*	*NpPMA1*	Plasma membrane ATPase 4	F	TCGTGGTCTGCGGTCATTAG
				R	TGCGAATGGTTTCTGCACTG
CotAD_23393	*GhPMA2*	*NpPMA2*	Plasma membrane ATPase 4	F	GGAGCCAACAGGCTTCAGAT
				R	AGCATTGCCAGCATTGTTTTCT
CotAD_69081	*GhHKT1*	*AtHKT1*	Sodium transporter HKT1	F	AGCAGGGAAGAGCATTTTGGA
				R	CGGGCCGGGATTAAAGAGAA
CotAD_03789	*GhVAP-c2*	*AtVAP-c2*	V-type proton ATPase subunit c2 (V-ATPase subunit c2)	F	AATCGATCGTCCCCGTTGTT
				R	GAGACCAGCTAGGCCACAAG
CotAD_08864	*GhVAP-c4*	*AtVAP-c4*	V-type proton ATPase subunit c4 (V-ATPase subunit c4)	F	GCCCATCTTTCATCCGGTCT
				R	AGCACCATACCTGACACCAG
